# 2019 ISCB Overton Prize: Christophe Dessimoz

**DOI:** 10.12688/f1000research.19220.1

**Published:** 2019-05-23

**Authors:** Diane Kovats, Ron Shamir, Christiana Fogg

**Affiliations:** 1 International Society for Computational Biology, Leesburg, USA; 2 Blavatnik School of Computer Science, Tel Aviv University, Tel Aviv, Israel; 3 Freelance Writer, Kensington, USA

**Keywords:** ISCB, Christophe Dessimoz, Award

## Abstract

Each year the International Society for Computational Biology (ISCB) honors the achievements of an early- to mid-career scientist with the Overton Prize. This prize was instituted in 2001 to honor the untimely loss of Dr. G. Christian Overton, a respected computational biologist and founding member of the ISCB Board of Directors. The Overton Prize recognizes early or mid-career phase scientists who have made significant contributions to computational biology or bioinformatics through their research, teaching, and service. In 2019, ISCB recognized Dr. Christophe Dessimoz, Swiss National Science Foundation (SNSF) Professor at the University of Lausanne, Associate Professor at the University College London, and Group Leader at the Swiss Institute for Bioinformatics. Dessimoz receives his award and is presenting a keynote address at the 2019 Joint Intelligent Systems for Molecular Biology/European Conference on Computational Biology held in Basel, Switzerland on July 21-25, 2019.

## Christophe Dessimoz: Learning to Stop Resisting

Christophe Dessimoz grew up in Switzerland and was deeply drawn to computers from a young age, right around the dawn of the web and widespread internet access. As a teenager, he spent a lot of time programming and developing websites. To avoid spending too much time in front of computers, he decided instead to pursue a first degree in Biology at ETH (Eidgenössische Technische Hochschule) Zurich, Switzerland. During and after his studies, he traveled and spent several months doing biology research at Northwestern University in the United States, Tsinghua University in China, and Chulalongkorn University in Thailand. Dessimoz recalled his struggles at the bench and feared he was just too clumsy to pursue a career in biology. He was also still drawn to computers, and he said, “I just stopped resisting,” and returned to ETH Zurich to complete a PhD in Computer Science under the mentorship of Gaston Gonnet.

**Figure:**
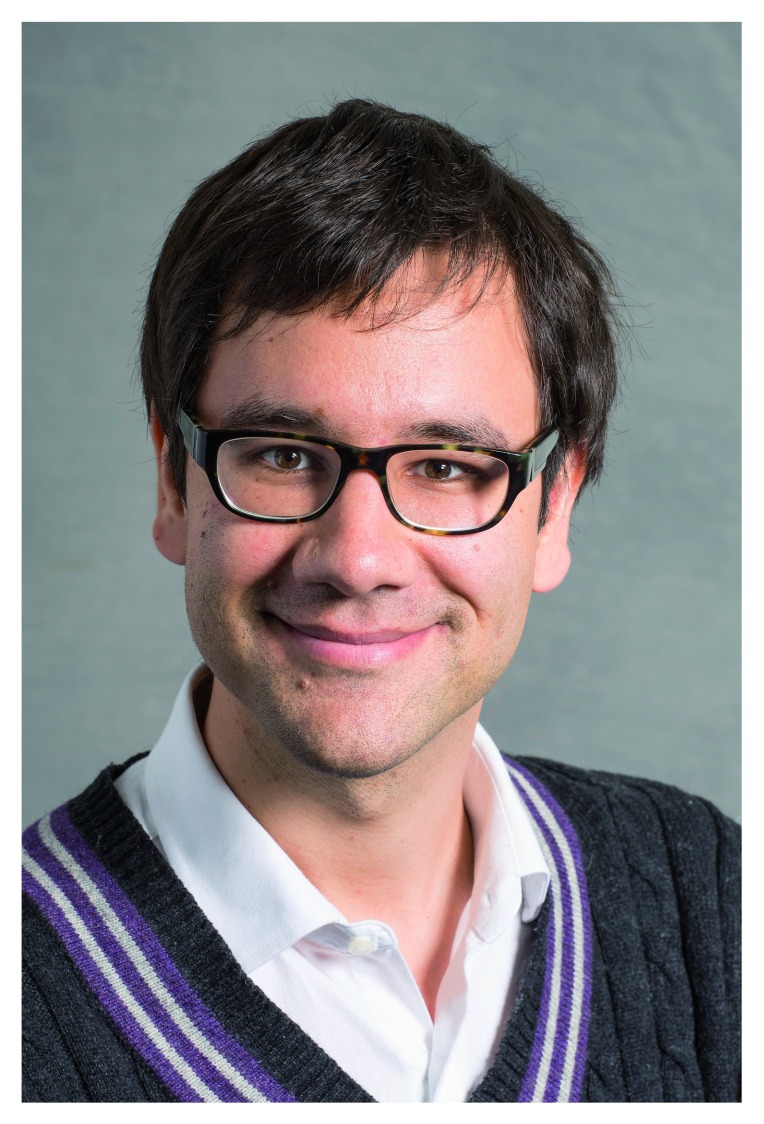
Christophe Dessimoz

Gonnet had expertise in symbolic and algebraic computation and helped develop a computer algebra system that became a foundation of the Maple system. Gonnet also cofounded the New Oxford English Dictionary Project, which created an electronic version of the Oxford English Dictionary that included a full text searching system. By the early nineties, this expertise in text searching and string algorithms led him to bioinformatics.

Dessimoz joined Gonnet’s team in 2004 to purse a PhD research project in comparative genomics, right at the time when genomes were starting to become routinely available. There was an obvious need to compare these genomes, and in particular to identify the corresponding genes in different species—the orthologs. Dessimoz’s first scientific paper introduced the “Orthologous Matrix” (OMA) algorithm. The work provided the foundation for the OMA database, which he kept working on ever since and which has become a well-regarded resource.

After completing his PhD and working as a teaching faculty member and senior research associate at ETH Zurich, Dessimoz received a fellowship from the Swiss National Science Foundation (SNSF) to be a visiting scientist at the European Molecular Biology Laboratory (EMBL)-European Bioinformatics Institute (EBI) in Hinxton, UK. He joined Nick Goldman’s group to collaborate on large-scale phylogenetic methods. During his stint at EBI, Dessimoz became increasingly interested in benchmarking and developed an appreciation for this often overlooked but critical methodological process. This interest is exemplified in his leadership role in the *Quest for Orthologs Consortium*, a community effort to improve orthology methods and their applications. Together with Adrian Altenhoff, his close associate since their days in Gonnet’s lab, Dessimoz developed a more direct approach to benchmarking orthology inference methods based on congruence tests between genes and species trees, which has become a definitive method for orthology benchmarking. He has continued to advocate for the importance of benchmarking and standardization for progress in bioinformatics.

In 2013, Dessimoz moved to University College London as a Lecturer and held a joint appointment between the Department of Genetics, Evolution & Environment and the Department of Computer Science. He was promoted to Reader in 2015, and he also joined the University of Lausanne (UNIL) as SNSF Professor. Dessimoz became a group leader at the Swiss Institute of Bioinformatics in 2015, and he continues to maintain his UCL and UNIL labs. Beyond his contributions to benchmarking, Dessimoz has made critical contributions to phylogenetics tests of sequence alignment accuracy. Despite the decades of work in this area, he said, “We still struggle to assess the correctness of alignments.” Dessimoz developed a novel assessment paradigm based on the plausibility of trees reconstructed from inferred alignments, and through this work, he showed that the common practice of inferring trees from “high-confidence” alignment columns only is counterproductive. Dessimoz has also made valuable contributions to methods used for Gene Ontology (GO) annotation, particularly with the development of a technique using historical time series to track the fate of inferred annotations over time. This approach has changed the way the GO consortium tracks annotation quality and has been adapted for other projects. Dessimoz continues to be drawn to research focused on evolutionary biology and orthology inference, and he also fascinated by the challenges of handling huge heterogeneous datasets.

Outside of the lab, Dessimoz has served the computational biology community in many capacities, including service on multiple editorial and advisory boards and conference committees. Dessimoz’s groundbreaking research has been recognized by several other awards, including the 2017 Sentinel of Science Award, 2016 (European Molecular Biology Organization) EMBO Young Investigator, 2015 Google Faculty Research Award, and 2012 Swiss Institute of Bioinformatics (SIB) Early Career Bioinformatician Award. Dessimoz feels deeply honored by his selection as the 2019 Overton Prize winner, and he acknowledges that the support and relative independence that his mentors gave, as well as the productive partnerships with his staff, trainees and collaborators have been instrumental to his success as an independent investigator. 

## Data availability

### Underlying data

No data are associated with this article

